# Issues and challenges in recruitment for government doctors in Gujarat, India

**DOI:** 10.1186/s12960-016-0140-9

**Published:** 2016-07-19

**Authors:** Bhaskar Purohit, Tim Martineau

**Affiliations:** Indian Institute of Public Health Gandhinagar (IIPHG), Sardar Patel Institute Campus, Drive in Road, Thaltej, Ahmedabad, 380054 India; Liverpool School of Tropical Medicine (LSTM), Pembroke Place, Liverpool, L3 5QA United Kingdom

**Keywords:** Gujarat, India, Medical Officers, Civil Service, Recruitment, Public Service Commission, Attraction and Retention, Human Resource Management

## Abstract

**Background:**

India faces a critical shortage of government doctors in rural and underserved areas. Several measures have been introduced to address the shortage, but significant problems still remain. The main aim of the current research was to understand the existing recruitment-related policies and systems in place for government doctors in Gujarat and to identify issues that prevent effective recruitment of doctors that could have implications for doctors’ shortage in the state. The research also aims to fill the knowledge gap in the existing literature on why recruitment in civil services is an important HR function to address the shortage of doctors.

**Methods:**

The study aimed at identifying the existing recruitment policies and practices for government Medical Officers (MOs) from Gujarat state in India. The analysis is based on document review to understand the existing policies, 19 in-depth interviews with MOs to understand the systems in place for recruitment of MOs, construction of job histories from interviews to understand various nuances in the recruitment system and five interviews with Key Informants to understand recruitment policies and their actual implementation. Thematic framework approach was used to analyse qualitative data using NVivo.

**Results:**

While the state has general recruitment guidelines called the Recruitment Rules (RRs), these rules are very wide-ranging and fragmented. The MOs were neither briefed about them nor received copies of the rules at any time during the service suggesting that RRs were not transparent. The recruitment system was considered to be slow and very sporadic having possible implications for attraction and retention of MOs. The study results indicate several other system inefficiencies such as a long time taken by the health department to provide salary benefits and service regularization that has a negative effect over MOs’ motivation. The study also found unequal opportunities presented to different categories of MOs in relation to job security, salary benefits and in recognizing their previous work experience leaving MOs unclear about their future thereby influencing the attraction and retention of MOs to government jobs negatively.

**Conclusions:**

If long-term solutions are to be sought, the health department needs to have an effective recruitment system in place with the aim to (1) address the slow and sporadic nature of the recruitment system (that is likely to attract more doctors and prevent loss of any doctors during recruitment) and (2) address the job insecurity issue that MOs have which also influences their other employment benefits such as salary, pension and recognition for the years of service they have given to the health department. Addressing these issues can improve motivation among doctors and prevent loss of doctors through voluntary turnover leading to better retention.

## Background

Health workforce has been identified as one of the most important resources to achieve effective health services. However, there is a severe shortage and inequitable distribution of health workforce in many countries with greater scarcity in countries where it is required most. India has been identified as one of the 57 countries with a critical shortage of health workers. Addressing the issue of shortage is important because the health systems of countries with a shortage of health workers are unable to offer even the basic health services to their population [1]. Empirical evidence indicates that an adequate size of health workforce is essential to achieve a minimum level of the health indicators [1, 2]. Among the health workforce working with the government health sector, doctors or Medical Officers (MOs) working with the Public Health sector are very crucial as they are the frontline health managers and service providers to the rural population. Despite many efforts in India to address the shortage of doctors in rural public health centres, India has largely failed to attract and retain MOs in rural health centres, and the shortage of doctors has been a matter of concern for many years [3, 4]. The Indian Public Health sector only employs at the most 10 % of total MOs [5]. Further, India also experiences a big variation in where the MOs are placed and distributed. Such differences exist between the states as well as within the states with the ratio of rural doctor to rural population far less than the ratio of total doctors to total population [5]. The overall figures for India suggest that there is a vacancy rate of nearly 21 and 42 % for MOs at Primary Health Centres (PHCs) and for specialists at Community Health Centres (CHCs), respectively, and a shortfall of 62 % for specialists at CHCs [6].

The public sector in Gujarat, where the current study was conducted, also suffers from a severe shortage of MOs and specialists, especially in rural areas. While the production of doctors in Gujarat has been sufficient to meet the shortages, very few medical graduates from the state actually join the government service which makes addressing the shortage of doctors a complicated issue. The state of Gujarat has taken various steps (explained below) to increase the availability of MOs and specialists, especially in underserved areas, similar to those used in many countries and across Indian states [7, 8]. Despite the use these strategies, the Public Healthcare System in Gujarat suffers from a severe shortage of doctors. Many medical graduates are trained in Gujarat. Currently, there are 19 medical colleges in Gujarat, both private and government. According to the latest data available from 2007–2008, the six government medical colleges in the state produced as many as 975 doctors with bachelor’s degree and a total of nearly 551 doctors with Post Graduate Degree in the year 2007–2008. This number increased to 1233 for medical graduates with bachelor’s degree and 595 for the medical post graduates in the year 2012–2013 [9, 10]. Nevertheless, according to the most recent available data, only 7 and 10 % of the medical graduates from government medical colleges appointed under the bonded category (a form of compulsory rural service) [7] actually joined the government service in the year 2004–2005 and 2005–206. This was explained by problems in the recruitment-related system such as legal hassles involved in bonds implementation and eagerness of medical graduates to pursue Post Graduate studies [11]. Similarly, 50 % of MOs appointed under a different recruitment categories (on an ad hoc category explained later) actually joined the government in 2004–2005 and 2005-2006 [12]. The vacancy level is 24 % for MOs at Primary Health Centres (PHCs) while the vacancy level is particularly high (77 and 93 %, respectively) for all specialists working with CHCs [6] (see Table 1 for details).

**Table 1:** Vacancy and shortfall of MOs at CHCs and PHCs in Gujarat

	Required (R)	Sanctioned (S)	In position (P)	Vacant (S-P)	Shortfall (R-P)
MOs at PHC	1096	1096	837	259	259
Surgeons at CHC	290	278	63	215	227
OB and GY at CHC	290	34	11	23	279
Physicians at CHC	290	0	0	0	290
Ped at CHC	290	34	5	29	285
Total specialists CHC	1160	346	79	267	1081

*Source:* Rural Health Statistics 2010

The Department of Health and Family Welfare or the Department of Health in Gujarat is headed by the Minister of Health and Family Welfare while the Principal Secretary of the Health and Family Welfare is the administrative head of the department and responsible for implementing the policies. There are various directorates under the Principal Secretary which are directly involved in implementation of various programmes and activities. The Department of Health and Family Welfare in the state has three directorates (Health, Medical Services and Medical Education) that are mainly responsible for technical as well as administrative support to the health-related activities in the state.

### Organizational structure and health system in Gujarat

The state of Gujarat is divided into six regions with all the 32 districts in the state falling under the six regions. Six Regional Deputy Directors (RDDs), one for each region, are in-charge for the health-related activities for the districts that fall under their region.

As per the state’s Civil Services Recruitment Rules 1967, the MOs working with the Health Department in Gujarat have been categorized into two classes, i.e. I and II. Both class I and II are gazetted posts [13]. Gazetted officers are government employees or public servants working at a professional/managerial/supervisorial level and have the authority to issue an official stamp.

At the district level, Chief District Health Officer (CDHO) is the overall in-charge of the Community Health Centers (CHCs) and the Primary Health Centers (PHCs) within the district. Several blocks or the administrative units constitute a district. Blocks are administered by the Block Health Officers (BHOs) who are MOs. Similarly, all the District Hospitals (DHs) within the district are headed by the Chief District Medical Officer (CDMO) of the District Hospital.

The public health delivery system in Gujarat has several tiers. At the top and district level is the DH which is a government hospital that caters to the health needs of the entire district providing mainly tertiary care. Next, at the block level, CHCs exist which are 30-bedded hospitals that constitute the secondary level of health care and provide referral as well as specialist health care to the rural population at the block level. CHCs cater to 80,000–120,000 population. According to health service norms, each CHC needs to be staffed with specialists as well as regular doctors or MO. One level lower to CHCs exist the PHCs that cover a population of 20,000 in hilly, tribal or difficult areas and 30,000 populations in plain areas with four to six indoor/observation beds. PHCs act as a referral unit for six sub-centres and refer out cases to CHC (30-bedded hospital) and higher order public hospitals located at sub-district and district levels. Each PHC needs to be staffed with at least one MO.

All graduate doctors are recruited as Medical Officers (MOs) in a class II position to work in Primary Health Centres (PHCs) and/or Community Health Centres (CHCs) whereas those holding a Post Graduate degree in clinical areas are recruited as specialists as class I. In addition to specialists, senior level positions at state, regional and district such as RDD, CDHO and CDMO are class I positions while the MOs working with PHCs and CHCs without Post Graduate specialization are class II positions.

### Recruitment and service-related terms

The state’s Gujarat Public Service Commission (GPSC) is responsible for recruitment of all gazetted posts including MOs. The main function of the GPSC is to conduct examinations for appointment to the services of the state and advises on the matters relating to methods of recruitment to various Civil Services of the state [13]. Medical staff in classes I and II in the state of Gujarat can be employed on four different types of contract: (1) ‘bonded’ contracts (for graduates from government training institutions); (2) ‘ad hoc’ contracts on indeterminate length; (3) fixed-term contracts; and (4) permanent contracts on passing the GPSC exam.

Under the compulsory rural service in Gujarat, all the medical graduates from the Government colleges enter the government service under the ‘bonded’ contract and have to sign a bond at the time of admission to medical college that requires them to compulsorily serve in rural areas for 2 years. For the bonded category, the government heavily subsidizes the tuition fee. Such candidates are required to join the rural service after they finish their medical internship. The bonded candidates do not have to go through any exam or interview as part of the selection process. The employment status of bonded contact doctors with the government remains temporary (meaning that they are not permanent employees of the government or gazetted officers) until they pass the Gujarat Public Service Commission (GPSC) exam. Once such MOs pass the GPSC exam, they are appointed as permanent employees of the government and are on ‘regular service’ and get ‘service regularization’ (explained below). This gives them a permanent and pensionable employment status. In case these MOs do not wish to serve the government after graduation, then they are required to pay the bond amount of Rs. 5,000,000 (USD 8,300). MOs are considered as civil servants after they clear the GPSC.

To address the shortage of MOs in the state, the Department of Health and Family Welfare in the past recruited MOs from Gujarat such as candidates from private medical colleges or outside the state. Recruitment of such MOs is called ‘ad hoc’ appointment and is temporary. Ad hoc MOs were required to pass the GPSC exam in order to be appointed as permanent employees. However, the contract of any temporary employee [or ad hoc MO] is terminated if they fail to pass the GPSC exam before the age of 45. Selection of ad hoc candidates is done through walk-in interviews that are conducted weekly either at the Commissionerate or at the RDD office. Currently, the government has stopped recruiting MOs in the ad hoc category. The MOs under the ad hoc category may not get employment benefits which are otherwise available to MOs who are on ‘regular service’.

A third type of employment contract is for 11 months and is referred to as ‘contractual appointment’. The contractual category includes all graduates from private medical colleges and MOs either from states outside Gujarat or MOs over the age of 45 who have not passed the GPSC exam. The MOs under contractual category do not get employment benefits which are otherwise available to MOs who are on ‘regular service’ such as higher salary, pension and promotion as per government rules. The years of service that contractual category MOs serve are not counted as government service which affect future salary level benefits ‘Tikoo grade’ (explained below) and pension benefits.

The central government in 1994 constituted a committee called Tikoo Committee to look into various salary- and promotion-related issues and to make the salaries of MOs at par with the central level. Since all the MOs who fulfill the requirement for promotion cannot be promoted due to limited availability of positions, a time-bound promotion based on length of service to MOs is provided which is equivalent to different senior level selection grades with accompanying increase in salary after 6, 13 and 19 years. In order to be eligible for a higher Tikoo grade, one needs to be on ‘regular service’. MOs who do not pass the GPSC exam and attain ‘service regularization’ do not get higher Tikoo grade irrespective of number of years served prior to passing the GPSC exam.

The GPSC prepares a list called ‘seniority list’ of all MOs in the state, and this list is handed over to the health department. This main use of the list is for promotion-related decisions. The MOs higher in the list are given preference for promotion. MOs who pass the GPSC exam before other MOs are ranked/placed higher on the list irrespective of total years served in the government before GPSC. However, if more than one MO appears for the GPSC at the same time, then the MO who scores better in the GPSC exam is placed higher in the ‘seniority list’.

### Civil services recruitment process

Historically, the recruitment of civil servants in many countries has commonly been through patronage [14] and is associated with higher levels of corruption and incompetent people being recruited [15, 16]. Hence, civil service reforms have focused on public administration to be free from corruption [17]. One of the most important aspects of civil service reform is merit-based recruitment which not only is conducive to economic growth and prevention of corruption [18] but also promotes more competent people getting into the system with less scope for corruption [19]. However, despite the advantages of having a recruitment systems in place to ensure recruitment of competent staff and prevention of corruption, some researchers report that progression based on merit through staffing agencies for civil services can be slow, rigid and complex [20].

### The importance of the recruitment

Recruitment is the first step in an employment cycle. The need to have right Human Resource (HR) policies and management at the core of Human Resource Management (HRM) systems for sustainable solutions to health system performance has been greatly emphasized [21, 22]. But what does HRM include? HR policies and management include several functions such as recruitment, placement and managing performance through appraisal systems [23–25]. Further, managing the workforce is a constant cycle of recruitment, selection, training and retention strategies. However, there is very limited research and information on the Human Resource Management (HRM)-related dimension and systems [26].

Recruitment is an important function of HRM that cannot be overlooked as it is the first step in matching the organization needs with individual needs [23]. Further, HRM practices including recruitment have been found to be a significant predictor of intention to leave in a business sector [27]. Recruitment practices have also been found to have positive effects on organizational commitment [28]. Understanding recruitment becomes particularly important as the limited research in the area of recruitment in civil services involving MOs suggests that the recruitment process involving Public Service Commission (PSC) can be very lengthy not only in India but also in other countries such as Malawi, Bangladesh and Nepal [29, 30].

### Conceptual framework for the study

Protracted recruitment processes can be a major demotivating factor among MOs affecting their attraction towards the government service and can negatively affect their final decision whether to join government services or not [9, 31]. Hence, the success in securing needed human resources for the civil service depends directly on public personnel recruitment practices as well as the ability of civil service to attract enough number of competent HR from the eligible pool [31].

An effective recruitment system therefore has two main purposes: (1) to attract people to the jobs through promises of what they get immediately and subsequently as benefits in the jobs [23] (the benefits people are likely to get also influence their decisions concerning whether to join or/and continue or quit their jobs having a direct influence over both attraction and retention) and (2) to select people with appropriate competencies and skills [23]. In a competitive labour market such as in Gujarat, doctors may prefer long-term benefits such as pension and job security provided in government jobs over immediate higher salary earning in the private sector. The conceptual framework for the study is based on the premise that the initial attraction of MOs to apply for, join and continue services with the government not only depends upon the long-term benefits that a government job offers but it also depends upon HRM-related recruitment policies and systems and how these policies and systems affect the long-term gains such as pension and job security.

The conceptual framework of the study is adapted from Boxall and Macky that refers to the importance of not only having the Human Resource Management (HRM) policies in place but also the way HRM policies are practiced, perceived and implemented [32]. The Boxall and Macky framework suggests link of Human Resource Management (HRM) performance causal chain with intended Human Resource (HR) practices, actual HR practices, HR practices as perceived by employees and employee reactions or behaviour [32]. Our study framework draws a link between intended recruitment policies and practices, MOs’ perceptions about these policies and practices, MOs’ reactions driven by motivation and its potential link to final attraction to the job as well as retention.

**Fig. 1:**
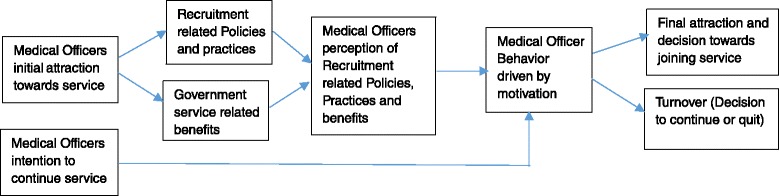
Conceptual framework for recruitment and how it is linked to attraction and turnover

The recruitment policies and systems could play an important role in influencing the key Human Resource for Health (HRH) outcomes such as turnover and performance. The health department in the effort of better HRH management may have certain policies and practices. These policies and practices may shape the positive or negative perceptions MOs have about recruitment. The negative or positive perceptions may further affect the MOs’ motivation (positively or negatively) having an influence over their behaviour leading to either decisions to join or continue the services affecting both attraction towards job as well as affecting the turnover (see Fig. 1).

Therefore, we argue that addressing the shortage of government MOs requires a greater understanding of the recruitment-related policies and systems. The main aim of the study described in this paper was to understand the existing recruitment-related policies and systems for MOs working with the government in the Department of Health, Gujarat, and to identify issues that hinder effective recruitment of doctors with negative consequences for MO staffing in the state. The study only included government doctors as the aim was to understand the effectiveness of government recruitment systems. The research aims to fill the knowledge gap in the existing literature on why recruitment in civil services is an important Human Resource Management (HRM) function with a potential to address the shortage of MOs in the state.

## Methods

### Study design

This was a qualitative study that included document review and interviews with Key Informants (KIs) to identify the policies and official procedures for recruitment. This was complemented by interviews with KIs and Medical Officers (MOs), and job histories, constructed from the data available through interviews, identify the actual recruitment practices. The study used qualitative methods as it was best suited to the scope of current study that aimed at assessing the recruitment-related policies, systems and perceptions of MOs that would not have been possible through quantitative study. Qualitative design also justifies the need for the study aimed at organizing the data into themes.

### Study setting

This study was conducted in Gujarat, India, in 2013. MOs working for the government health department placed at rural health centres from three different districts from the state were included in the study. The districts were purposively selected. Based on initial discussions with several MO and state level officers (outside the study), a list of a few desirable, not so desirable and not at all desirable districts for MO posting was made. As several districts were identified in each of the above category, three districts meeting the above criteria were selected from three different regions from the state for a larger geographical representation.

### Data collection methods and sampling

#### Document review

Document review was carried out to understand the recruitment-related rules and policies. The review included the Civil Services Rules and the RRs for various cadres of MOs in the state as detailed under various government orders. Documents relating to service conditions such as job descriptions were excluded.

#### Interviews with KIs

This group included informants who occupied key state and district level positions purposively selected for their knowledge of the study topic and to gauge their opinions on the existing policies and systems relating to recruitment. The study included five KIs to ensure that the views and perspectives of a range of stakeholders on study topic could be represented. A number of interviews with KIs were conducted until ‘saturation’ was reached—that is ‘new data no longer shed new light’ [33].

Three out of five interviews with KIs were conducted in Hindi (the main language spoken in India) and two interviews in English as the two KIs preferred to give interview in English using topic guides. The main focus of interviews with KIs was to understand the recruitment-related policies and rules, systems involved in recruitment, perceptions of KIs on effectiveness of existing recruitment-related policies and strategies to address the shortage of MOs and recommendations for further improvement. Saturation was reached after five KIs were interviewed; hence, no more interviews with KIs were conducted after the five.

#### Interview with MOs

This group consisted of class I and II MOs who were the main subjects of the study and included MOs from PHCs and CHCs as well as the BHOs. The study used purposive sampling to ensure representation of MOs from three different districts (representing three different geographical regions) from the state. A number of interviews with MOs were conducted till the time saturation in information was experienced. In total, 19 interviews were done with the MOs that included three female MOs.

In-depth interviews with all the 19 MOs were conducted in Hindi (national language of India which is widely spoken and understood across India). The main objective to interview was to explore MOs’ knowledge, perceptions and understanding and their experiences of recruitment-related systems.

All interview recordings with KIs and MOs were transcribed verbatim and then translated into English text by the primary researcher (BP). Written consent was sought from study participants, and the interviews were audio recorded. Important notes relating to job history were also taken during the interviews.

#### Job histories

During the interviews with MOs, brief job histories were constructed from the data available through in-depth interviews with MOs to get deeper insights into recruitment systems.

### Data analysis

#### Document review analysis

Simple content analysis of the documents was done to understand the recruitment policies and rules. This included the main policy document called Civil Service Rules and various Recruitment Rules as detailed under various government orders.

#### Analysis of interviews

Interviews were analysed using a thematic framework approach which is a matrix-based method to arrange and synthesize data [34]. The framework analysis approach was best suited to the scope of current research as the aim of the research was to present themes identified in the data. The framework approach was used to identify key words, themes and sub-themes that emerged from the 24 transcripts, and the results of the study are reported against the key themes and sub-themes. The transcripts of the 24 participants (KIs and MOs) were coded and grouped according to the themes and sub-themes identified based on a priori and the emergent codes from the data. A detailed analysis was performed using NVivo on the transcribed texts [35].

#### Analysis of job histories

Simple descriptive statistics derived from the interviews were used to calculate the average time it took for job regularization for the MOs.

### Research ethics

The ethical approval for the study was sought from institutional ethical review committee at the Indian Institute of Public Health Gandhinagar (IIPHG). Relevant permission for the study was also obtained from the Department of Health, Government of Gujarat and the Commissionerate of Health. Written consent was obtained from all the MOs and KIs. The participation in the study was completely voluntary, and respondents were assured of anonymity at all times of the study.

## Results

We first present the demographic profile of the study respondents. Next we explain the MOs’ knowledge about the Recruitment Rules (RRs). In the final part of the ‘Results’ section, we explain the implementation of the RRs by explaining in details the systems of recruitment, issues identified by the study respondents in the recruitment systems and suggestions from the respondents to address the issues identified under recruitment.

### Demographic details

The demographic profile of the study respondents is presented in Table 2. Although the study used purposive sampling to try and maintain gender balance by including a good number of Lady Medical Officers (LMOs) (specific term used in India for female MOs), the overall availability of LMOs was low and the present study could only include three LMOs out of 19 total respondents. However, there was a balance of ad hoc and bonded MOs with an almost equal representation of both the categories in the study.

**Table 2:** Distribution of MOs based on demographic and work profile

Gazetted officer	District 1	District 2	District 3	Total
Class I	1	1	1	3
Class II	4	5	7	16
Gender				
Male	5	5	6	16
Female	0	1	2	3
Entered service through				
Bonded	3	4	4	11
Ad hoc	2	2	4	8
Place of work				
PHC	1	3	3	7
CHC	0	2	2	4
SDH/DH	3	1	0	4
BHO	1	0	3	4

### Recruitment rules and policies

The study findings suggest that the state does not have any specific Recruitment Policy for MOs. What exist in the name of Recruitment Policy are the some Recruitment Rules (RRs) which are overall governed by Civil Services rules. The RRs for staff in the Health Department of Gujarat are regulated by the Gujarat Civil Services Classification and Recruitment (General) Rules 1967. These rules have undergone modifications from time to time based on amendments [11, 13].

The study found that the RRs for different categories of MOs are fragmented which means that they do not exist in one single document, and such rules have undergone changes from time to time. The RRs for different cadres of doctors are laid down in a form of notifications that are usually three to four pages long.

Based on document review and interviews with KIs, it could be concluded that the RRs are very broad and consist details like qualification required and experience required and promotion-related details such as ratio between promotion and direct selection for different positions of MOs.

I would say that there is no recruitment policy, but there are policy documents which are fragmented. These fragmented documents relating to recruitment and transfers are considered as policies. These documents may not give complete idea about the policies but they give some idea. But there is no complete, comprehensive policy statement. There are recruitment-related rules called RRs that talk about the qualification, experience, ratio between promotion and direct selection, process of how ad hoc need to be appointed, how MOs need to be confirmed on long term basis after clearing GPSC etc (KI 1).

### Knowledge relating to recruitment rules/policy

Most of the MOs knew only about two aspects of the recruitment rules: (1) various categories under which MOs are recruited, i.e. bonded, ad hoc and contractual appointments and (2) the actual system and steps involved in recruitment and selection followed for different categories under which MOs are appointed. The responses of the participants indicated that their overall knowledge about RRs was generally low.

I know this much that every Monday there are interviews and that one can walk-in and give interviews, and then they will give you placement (MO 11)

Only one MO reported understanding about the RRs beyond the two aspects presented above where the MO described some of the promotion rules that are included in the RRs.

The recruitment rule is such that once an MBBS gets an experience of working with a PHC or CHC, he then enrolls for Diploma in Public Health. So after that his experience plus Diploma in Public Health, qualifies him for Class 1 with an experience of Health for 5 yrs is a must (MO 17)

All the MOs in the study reported that they were not given a copy of RRs anytime during their service nor were ever briefed about the RRs. The importance of knowing RRs was reflected by one of the MOs who suggested that lack of awareness about the rules can delay the service-related benefits MOs are eligible for.

No one told me anything about the RRs (MO 9)

I did not get any rules from anyone and I did not even know about Tikoo grade. So when I got to know about it, I enquired at the state headquarters that it’s been 6 yrs and I am due for the benefit. So from there I got to know that my Confidential Reports (CRs) are missing (CRs are important for the decision to be made in this regard) (MO 11)

Since most of the MOs did not know about the RRs, one of the KIs was of the opinion that the RRs are not transparent and such rules are not publicly available on the government website.

The RRs are not transparent. See after 20 years of service, I have got some set of rules or a copy of rules. There are doctors who have not thought of joining government service because they always wanted to do private practice or Post Graduate. But tomorrow when they enter Government service, they know nothing about the RRs. These rules are not available on the website. So there is no transparency (KI1)

### Implementation of RRs and policies

According to the study respondents, the MOs in the bonded, ad hoc and contractual categories may be recruited at two stages, one before GPSC which is an initial recruitment and the other at the time of GPSC (which could be through direct recruitment or promotion). Since the recruitment system before GPSC for the three categories is different, it is briefly explained below. However, the recruitment system is the same for all categories of MOs during the GPSC.

#### Bonded category

The KIs and MOs indicated that there is no formal recruitment and selection system followed for bonded candidates when recruiting them directly from the government medical colleges. Every bonded candidate irrespective of marks scored in the Government Medical College is recruited and placed in the Government Health Centers in rural areas as a bonded candidate.

MOs under the bonded category usually receive an order from the health department during the final year of their MBBS studies (at the time of internship) that contains the details of their first posting with the government. According to the MOs, the time it took for them to receive such order varied significantly. While in most cases MOs responded that they received their orders during the final month of internship, however, in few cases, MOs responded that they received such orders several months later after completing their MBBS. According to one of the MOs, the delay in receiving orders can influence the decision of MOs whether to join the government or not. However, no data was available to explain the reasons why bonded doctors received their orders in varied periods.

There was no interview and they took me directly as bonded candidate. After the internship [MBBS internship] a list is prepared and bonded posting is made based on that list (MO 5)

As soon as bonded MO finish the internship, they should receive the order. Ideally one should receive such orders at least 2 days before finishing the internship. However such orders are received after 3–6 months of finishing the internship. If the health department can send the orders just before the completion of internship, at least 5 % [of the bonded MOs] would join. The health department sends such orders after 6 months and in this process they are not even aware how many of the Medical graduates have enrolled for Post Graduate (MO 17)

Another issue raised by MOs was ineffectiveness of bonds in order to make the MOs serve in rural areas and the small amount required to be paid to relieve oneself from the bond.

Although the bonded candidate are given their choice of location for posting but still the bonded candidates don’t join. And the reason for not joining in Gujarat is that they can pay the bond amount of 75 thousand rupees [USD 1250] so if they have money, they pay the amount. Only 20 % of the bonded doctors join the government while others pay the amount and don’t join. So this is one of the reasons for the shortage of doctors (MO 11)

Another issue indicated by MOs was lack of any system in place to notify the MOs about available vacancies they can opt to while getting required and placed after finishing their MBBS.

I made my own effort to find out about available vacancies and that’s how I got XYZ [my current place of work] (MO 2)

Due to low number of MOs joining the government service under the bonded category, it was suggested that certification of registration as a doctor by a medical council must only be provided to MOs (under the bonded category) after they complete 3 years of compulsory rural service. Yet another recommendation was to reduce the bond period from 3 to 1 year.

### Ad hoc category

According to study respondents, the MOs under an ad hoc category appeared for walk-in interviews that were held at all the six RDD regions. As far as the system of selection was concerned, the MOs responded that the interview panel included RDD along with some officials from the state health department.

I joined in 2004 November and the interview took place at [place x]. It was a walk-in interview taken by RDD. The interviews are held every Monday (MO 1)

Two issues were brought up by the MOs relating to the recruitment system of the ad hoc category that may have influence attraction and actual number of MOs who join the government. First is that sometimes the system is slow and second is that there is an absence of a system in place to notify the ad hoc MOs about the available vacancies in the region.

The recruitment process for Class 2 particularly for ad hoc category before GPSC is lengthy as well as complicated. Although on papers it is walk-in interview but it’s nothing like that you walk-in and you get a choice of your place the next day. The file keeps moving from one place to another. For class 2 officer the health department does not revert back for 2 months, so in the meanwhile the MOs figure something else out (MO 17)

The health department haven’t developed a system yet, that a person walks in and is asked for his choice of location. Only if there is a vacancy then the person is given his choice. Hence either they leave after joining or don’t join at all (MO 17)

I managed on my own to find about the vacant positions (MO 7)

### Contractual category

As the study did not include any MO from the contractual category, the study did not document the detailed system involved in recruitment and selection of contractual MOs. However, several recruitment- and service-related issues relating to contractual MOs were brought up by the KIs and MOs.

According to one KI, the current policy of the government to recruit MOs under a contractual category causes a lot of inconvenience to such doctors as the contract needs to be renewed periodically. Further, such a strategy does not assure them that they will remain posted at their previous work place/posting. According to the KI, this is one of the reasons for dissatisfaction among the contractual MOs and may lead to a high turnover. One of the other drawbacks of working as a contractual MO was that the work experience with the government (prior to GPSC) is not counted towards service continuation which affects the benefits such as job security, promotion and other salary-related benefits.

There are problems in contractual appointments. Problem in the sense that first the posting used to be for 11 months, then in between there were orders that the posting will be for 6 months. The main problem is that the renewal process happened every 6 months or after 11 months. If someone is with a PHC or CHC, then after 6 months he will be transferred. Then the person will be sent to state headquarters, and then he will be removed from there and then will be sent elsewhere and then back to headquarters. The file [for recruitment and posting] keeps moving from one place to another. This person will get his salary for 6 months, and then he will be without salary for 2 months [till new appointment is given] and so on. So the doctor goes through a lot of inconvenience. So such a doctor will go through transfers one or two times and the third time he will resign (KI 2).

The work experience of the contractual is not counted in service continuation (MO 19)

Recognizing the recruitment issues involved in the contractual category such as insecurity of job and lack of employment benefits that are otherwise available to MOs in the regular service, MOs and KIs suggested that rather than renewing contracts every 11 months, a system should be developed that allows contractual MOs to be recruited on a probation period, and on successful completion of the probation period, these MOs must be given a permanent employment status without the involvement of GPSC.

### Recruitment through GPSC

The recruitment under GPSC is open to ad hoc, bonded and contractual candidates provided they are within the age limit. Once the candidates under the bonded category complete the bond, they are eligible to appear for the GPSC exam. Similarly, MOs appointed under ad hoc and contractual categories are free to appear for the GPSC interviews whenever such exams/interviews are offered.

Several issues were indicated by the KIs and MOs with the recruitment system under GPSC. These issues were as follows: (1) Periodicity of the GPSC exam that delays the MOs’ service regularization and may reduce the scope for MOs to be eligible for GPSC as they cross the age limit required for GPSC and (2) a slow recruitment system under GPSC that may influence the decision of MOs whether to work with the government or not. The quotes given below suggest that the sporadic nature and slow recruitment system under GPSC may influence MOs’ decision negatively to join the government, or some MOs may lose interest in the job during this long system and may decide to either not join the government or join the private sector or start private practice instead.

GPSC is not regular, it is announced once in 8-10 yrs. The system is lengthy as well as complicated for Class 2 MOs particularly (MO 17).

The process of recruitment right from when government sends requirement to the GPSC to the time candidates are selected can take at least 9 months to 12 months….. And definitely in the meanwhile MOs get some another jobs and those candidates who are willing to join the government will not wait till their appointment order comes (KI 4)

Unless and until the person has a regular appointment he or she won’t be interested in continuing the job. Despite repeated request from government side to GPSC, conducting GPSC is a difficult task. The GPSC exam is not happening regularly. So the person who is serving for 11 months may not like to serve in the remote part unless their job is secured. (KI 3)

The job histories extracted from the interviews with MOs suggest a gap from 1 year to as long as 11 years between the time candidates joined (both ad hoc and bonded categories) the government service to the time the candidates appeared for the GPSC exam. According to most of the MOs, such a huge gap was primarily because the GPSC exam was not offered during this period (see Table 3 for details).

**Table 3:** Distribution of respondents according to various demographic and work-related variables

Respondent	Gender	Age	Class	Category	Total years of experience	No. of years between joining the service and passing the GPSC exam^a^
1	M	36	2	Ad hoc	9	1
2	M	35	2	Bonded	10	1
3	M	53	1	Bonded	22	1
4	M	43	2	Bonded	10	5
5	M	42	2	Bonded	15	7
6	M	31	2	Bonded	7	6
7	F	39	2	Ad hoc	13	6
8	M	46	2	Ad hoc	16	1
9	M	51	1	Ad hoc	21	3
10	F	30	2	Bonded	4	1
11	M	44	2	Ad hoc	11	4
12	F	32	2	Bonded	4.5	3
13	M	42	2	Bonded	18	11
14	M	40	2	Ad hoc	8	3
15	M	32	2	Bonded	8	1
16	M	54	2	Ad hoc	30	NA
17	M	37	2	Bonded	10	4
18	M	55	1	Bonded	29	8
19	M	36	2	Ad hoc	10	5
Average		40.94			13.44	3.94

^a^The gap between the time when MOs (either ad hoc or bonded) first joined the government service and the time they were confirmed in the government service through GPSC. As explained through the table, this gap was fairly large in the case of most of the MOs

The very few MOs who passed the GPSC exam within their 1 to 2 years of joining the service considered themselves lucky that the GPSC exam was offered within a reasonable time after they joined the service.

I was very fortunate that I got my GPSC examination very soon after my joining and I cleared that (MO 3).

One of the other issues that KIs and MOs indicated is that the Medical Services/Health and Medical Education departments were water-tight meaning that a shift of MO from Medical Education to Public Health and vice versa requires passing the GPSC exam again. For example, if a MO (who is part of the Public Health Department) wants to be a tutor at a medical college (part of the department of Medical Education), then such a MO needs to clear GPSC exam again for Medical Education and vice versa. Yet another issue which was pointed out was that the prior experience in the other department (Medical Education or Health Department) is not counted towards service continuation if the person moves to another department.

I voluntarily got myself transferred because the experience in Medical College is not counted in Health. So the 3 yrs I spent in the Medical College is gone a waste and does not count towards my experience (MO 17).

The various department within health (Medical Education and Public Health) are water tight compartments. Suppose an MO has worked with a PHC for 5 yrs and now he wants to join as a tutor then he has to appear in GPSC again. In this process if he crosses the age to 35, then he has to apply through the department and get an NOC from the Medical College and only then he can appear for GPSC (KI 1).

In light of the issues concerning GPSC, several suggestions were given by the KIs and MOs. One of these was to create a separate medical board that looks into the recruitment of MOs. One common suggestion was the need to conduct the GPSC exam regularly as it can prevent dissatisfaction that many contractual doctors have about getting their contact renewed every 11 months.

Regular appointments through GPSC will help us a lot. If we have GPSC on yearly basis, then it can prevent the dissatisfaction and frustration of many MOs who join the services in ad hoc and contractual category for 11 months (KI 2)

The only problem with recruitment is GPSC but they highly over-burdened as they are doing recruitment for all gazetted posts which are in thousands of numbers. Naturally GPSC cannot cope with this kind of challenge, so we have proposed a separate recruitment board which may be called Medical recruitment Board (MRB) (KI 3)

### Issues with service continuation, regularization and Tikoo grade

As mentioned in the ‘Results’ section, the matters relating to service continuation, regularization and Tikoo grade can be very important to MOs. These issues closely relate to what implications recruitment systems and rules may have on employment benefits such as getting a higher grade (Tikoo grade) depending on the number of years the MO has been on regular service, whether work experience of a MO (prior to GPSC) is counted towards service continuation which can impact the Tikoo grade.

While most of the MOs indicated that their period of service before GPSC was counted towards service continuation, the process of applying and getting service continuation can be cumbersome and very slow. However, there were MOs who reported that they did not get service continuation despite being long due for service continuation.

Although I have been confirmed by the GPSC in 2007-08, I haven’t received any letter for Seniority, nor for Service continuation. The government needs to give a letter for service continuation from my date of joining but they haven’t as yet. The last seniority list prepared is updated till 2007 and my name is not in the seniority list (MO 11)

I have completed 18 years and eligible for two Tikoo grades but I have not got even one… There are so many Doctors whose 2nd Tikoo is pending. The health department has no idea how much are we at loss. If you calculate, we get Rs. 10,000 [USD 170] less every month (MO 13)

## Discussion

This was an exploratory study aimed at understanding the recruitment-related policies and system for MOs and its implications for improved recruitment in the state that could potentially address the shortage of MOs in the state. Although the study aimed at throwing light on recruitment-related systems, the study has several limitations. Because of the wide-ranging nature of recruitment rules and system in how the government defines recruitment (that often includes cross cutting issues such as promotion, service regularization, Tikoo Commission), it was difficult to separate out these issues from recruitment. Secondly, it was beyond the scope of current research to assess actual implementation of all aspects of recruitment-related rules such as promotion. Hence, the system audit to understand the actual recruitment practices is confined only to the recruitment and selection system that took place before and during the GPSC exam for MOs. Since the RRs were not easily available and were very fragmented, the document review for the current study is based on a limited set of documents that was available at the time of study. More in-depth understanding of recruitment system and how such system links with other HR systems such as salary, promotion and other service benefits is a subject for further exploration. As the study was conducted only in Gujarat based on views and experience on 24 respondents, the results cannot easily be generalized across the states and nationally.

The task of locating and identifying the recruitment rules and policies was challenging because of the absence of clearly laid down rules and policies in one place. Although RRs exist, they were not available and accessible under the public domain such as government websites or any other government repository. In the absence of availability and access of RRs to the MOs either on the government website or in other ways such as booklets, the RRs were perceived to be non-transparent as most of the study respondents did not know about the rules. The study findings suggest that if the MOs do not know the rules then they are also not likely to know certain future benefits, which influence their behaviour and affects attraction and retention. Also, the RRs were present in a very fragmented form and were wide-ranging that included serviced-related matters such as promotions. This reflects the complexity and the difficulty to manage such rules which was also indicated by one of the KIs. Since the RRs contain important details about several service-related rules, it is important from an HRM perspective that such rules are made available to MOs, especially in the absence of any formal induction programme in place for MOs in Gujarat. Transparency relating to HR policies in Civil Services is now an important issue, and many Indian states and several other countries that follow Civil Services Rules for recruitment have such rules available on the their websites. Although RRs often cover issues relating to promotion and transfer, an attempt should be made to separate these out for a better understanding and implementation of HR-related functions.

The study indicates several system inefficiencies such as a long time taken by the health department to provide Tikoo grade (salary benefit) and service regularization (job security). Such system inefficiencies cause inconvenience to the MOs and may affect their motivation negatively. In a few cases, the MOs working on an ad hoc basis for long duration were denied service-related benefits. This was found to be an important reason for demotivation, which is not only indicated in the current study but also corroborated by another national study with MOs in two Indian states [9]. The current study also suggests that the recruitment system under GPSC was infrequent and can be very slow similar to that of other states in India such as UP [36] and other countries such as Bangladesh and Nepal [37, 38]. For example, recruitment through PSC in Nepal can take almost a year having implications for frequent occurrence of extended vacancies for frontline health service provider positions [38]. The slow recruitment system by GPSC has been suggested as a main reason for the shortage of MOs in Gujarat [9]. Similarly, recruitment under the Civil Service Commission in Malawi is not only lengthy (that can take at least 6 months from the time a post is advertised before it is filled) but also expensive for the government with the average cost, in replacing one professional officer, of $74,504 between 1990 and 2000 [29]. However, the ineffective recruitment systems in civil services not only involve high cost for employers or the government, the study results also indicate that MOs have to pay even higher cost because of such laggard recruitment systems. This sporadic nature of GPSC creates a long gap between the time MOs first join the government service and the time they pass GPSC exam that has implications over service-related benefits such as seniority list and service continuation (important for promotion and recognition of work MO has put through years), service regularization (job security) and Tikoo grade (higher salary), which are all found to be important factors of motivation among Indian government MOs [39].

The issues brought up in the study relating to service-related benefits were a cause of concern and demotivation for MOs as the inefficiencies in such a system creates certain parity issues with salary, seniority and other benefits mainly due to infrequency of the GPSC. Such parity issues have been also reported in a study with MOs in the state of Madhya Pradesh, India [9]. Hence, one of the possible solutions to the problem of infrequent GPSC could be lateral recruitment from outside the civil service, but such recruitments must be on a regular basis and must ensure employment-related benefits such as job security and pension to the MO. Recruitments through lateral entry will not only reduce the burden on GPSC but can also make the system or recruitment more frequent as has been initiated in China [40] as well as Haryana, India. The study results also indicate that slow recruitment systems and their implications for other service-related benefits may create a negative feeling among MOs about their job security, the most important motivation factor found among India MOs working with the government [39]. Lack of motivation and frustration with the current system further affects the HR levers of attraction and retention making the shortages even more severe.

The study also found difference between the recruitment and selection process for MOs appointed on contractual and ad hoc basis. While the MOs appointed on ad hoc basis have to go through a walk-in interview, there is no selection process followed for the bonded candidates before GPSC. One possible explanation for having no selection process for bonded category is the very limited number of MOs who join the government against the huge vacancies, and if the selection process is in place, it may further limit the MOs who join in the bonded category. However, this puts a question mark against the whole idea of competence-based or merit-based recruitment, identified as the most meritocratic way of recruitment [41].

One of the most concerning aspects of recruitment from an HRM point of view was the unequal opportunities presented to different categories of MOs (such as ad hoc, bonded and contractual) in relation to job security (regular service), salary benefits (Tikoo grade) and recognizing their previous work experience and efforts (service continuation) despite being recruited for similar positions and with similar educational background. Studies done with civil servants in Bangladesh also report such discrepancies [30]. The study results clearly indicate that the contractual category is the worst affected in this matter and it was clear from the study that such recruitment is a deliberate strategy so that the government can get away from paying benefits to contractual staff that are otherwise available to regular MOs as the contract model is used with the objective of reducing government expenditure [42]. Clearly, the MOs recruited under such a category never feel secured about their job and have the fear of being discontinued from the job anytime. Such issues can create demotivation and frustration among those in the contractual category, and currently, with no appointments of doctors in the state in the ad hoc category, and with very less joining in the bonded category, the government really needs to look how the recruitment under the bonded category can be made effective to address the issue of attraction and retention of MOs appointed under this category. A study done in India with government health staff found that job satisfaction is higher among regular staff compared to contractual staff, especially in relation to privileges and facilities relating to the job and career development [43]. The issues identified under the study are contrary to some of the characteristics of good HRM systems with emphasis on providing employment security, selective hiring of new personnel and reduced status distinctions and barriers [44]. As reflected in the study findings, the slow recruitment and posting systems were also a cause of concern among MOs in the ad hoc category which suggests that same problems may persist in the system of recruitment for the contractual category. Literature from other countries such as Nepal supports the current study findings that regulations such as limiting the duration of temporary contracts and the lengthy duration of the contracting system (reportedly up to 5 months in Nepal) are important contributing factors to high turnover among contractual staff [38].

## Conclusions

There is a great need to address the shortage of MOs in the government health institutions of Gujarat. While production of more doctors and regulatory measures such as compulsory rural service could be possible solutions to the problem, they do not address some of the fundamental recruitment problems identified by the study that greatly influence the availability of MOs in the state. Some of the issues identified under the study are not confined to Gujarat. These problems are in many respects similar to those in many Indian states [36] and other developing countries such as Bangladesh and Nepal [30, 37, 38].

The current study identifies several issues in the system of recruitment and with other HR systems such as employment benefits and job security which are found to be closely related to recruitment policies and systems that can have potential contribution to MOs’ shortage and turnover in the state. Hence, if long-term solutions are to be sought, the Department of Health needs to have an effective recruitment system in place with the aim to (1) address the slow and sporadic nature of the recruitment system (that is likely to attract more doctors and prevent loss of any doctors during the recruitment system) and (2) address the job insecurity issue that MOs have which also influences their other employment benefits such as salary, pension and recognition for the years of service they have served the health department. Addressing these issues can improve motivation among doctors leading to better performance, and prevent loss of doctors through voluntary turnover leading to better retention.

Given that there is no alternative to recruiting competent MOs and retaining the existing and the new recruits with higher motivation, the problems identified through this exploratory research must be addressed. This would require the Health Department to take a more holistic perspective of the process and consequences of recruitment. From a strategic HRM perspective, attraction, retention and effective management of HRH is really important for better availability, distribution and management of doctors. In addition, to understand the full impact of current recruitment practices, we suggest larger scale research, possibly using survey data, in Gujarat and other states. It may also be useful to carry out research for other contractual cadres who practice Ayurveda and homeopathy (alternative systems of medicine). Such contractual staff have been widely recruited throughout Gujarat under the contractual category and in other states in India to fill the gap in shortages of MOs, especially at PHCs.

## Abbreviations

BHO, Block Health Officer; CDHO, Chief District Health Officer; CDMO, Chief District Medical Officer; CHCs, Community Health Centres, DH, District Hospital, GPSC, Gujarat Public Service Commission; KIs, Key Informants; MOs, Medical Officers; PHCs, Primary Health Centres; PSC, Public Service Commission; RDD, Regional Deputy Director, RRs, Recruitment Rules
